# Equity and Generalizability of Artificial Intelligence for Skin-Lesion Diagnosis Using Clinical, Dermoscopic, and Smartphone Images: A Systematic Review and Meta-Analysis

**DOI:** 10.3390/medicina61122186

**Published:** 2025-12-10

**Authors:** Jeng-Wei Tjiu, Chia-Fang Lu

**Affiliations:** 1 Department of Dermatology, National Taiwan University Hospital, National Taiwan University College of Medicine, Taipei 100, Taiwan; 2 Grace Smile Dental Clinic, Taipei 100, Taiwan

**Keywords:** artificial intelligence, dermatology, diagnostic accuracy, skin cancer, dermoscopy, smartphone imaging, fairness, skin tone, generalizability, meta-analysis

## Abstract

*Background and Objectives*: Artificial intelligence (AI) has shown promising performance in skin-lesion classification; however, its fairness, external validity, and real-world reliability remain uncertain. This systematic review and meta-analysis evaluated the diagnostic accuracy, equity, and generalizability of AI-based dermatology systems across diverse imaging modalities and clinical settings. *Materials and Methods*: A comprehensive search of PubMed, Embase, Web of Science, and ClinicalTrials.gov (inception–31 October 2025) identified diagnostic accuracy studies using clinical, dermoscopic, or smartphone images. Eighteen studies (11 melanoma-focused; 7 mixed benign–malignant) met inclusion criteria. Six studies provided complete 2 × 2 contingency data for bivariate Reitsma HSROC modeling, while seven reported AUROC values with extractable variance. Risk of bias was assessed using QUADAS-2, and evidence certainty was graded using GRADE. *Results*: Across more than 70,000 test images, pooled sensitivity and specificity were 0.91 (95% CI 0.74–0.97) and 0.64 (95% CI 0.47–0.78), respectively, corresponding to an HSROC AUROC of 0.88 (95% CI 0.84–0.92). The AUROC-only meta-analysis yielded a similar pooled AUROC of 0.88 (95% CI 0.87–0.90). Diagnostic performance was highest in specialist settings (AUROC 0.90), followed by community care (0.85) and smartphone environments (0.81). Notably, performance was lower in darker skin tones (Fitzpatrick IV–VI: AUROC 0.82) compared with lighter skin tones (I–III: 0.89), indicating persistent fairness gaps. *Conclusions*: AI-based dermatology systems achieve high diagnostic accuracy but demonstrate reduced performance in darker skin tones and non-specialist environments. These findings emphasize the need for diverse training datasets, skin-tone–stratified reporting, and rigorous external validation before broad clinical deployment.

## 1. Introduction

Artificial intelligence (AI) has rapidly emerged as a promising tool for supporting dermatologic diagnosis, particularly in the classification of pigmented and non-pigmented skin lesions. Landmark advances in convolutional neural networks and deep learning have demonstrated dermatologist-level performance in melanoma classification [1], while more recent multimodal and transformer-based architectures continue to improve diagnostic accuracy across diverse disease presentations [2]. With the growing availability of clinical, dermoscopic, and smartphone-based images, AI technologies have increasing potential to complement dermatologists’ workflow and broaden access to early skin-cancer detection [3].

The clinical need for reliable diagnostic support tools is substantial. Skin cancer represents one of the most common malignancies worldwide, with more than 3.4 million cases of non-melanoma skin cancer and over 325,000 new melanoma diagnoses annually [4]. Early detection significantly reduces morbidity and mortality, yet timely access to dermatologic evaluation remains uneven. Many low- and middle-income countries experience severe shortages of trained dermatologists, with ratios often below one per 100,000 people [5]. Even within high-income regions, dermatology services show significant urban–rural disparities, leading to delayed diagnoses and heavy reliance on primary-care or teledermatology consultations [6]. These structural limitations have motivated the development of scalable AI systems capable of supporting lesion assessment outside specialist environments.

Despite rapid technical advances, questions remain regarding the generalizability, equity, and clinical reliability of dermatology AI systems. Many models are trained on narrowly curated datasets that may not reflect real-world variation in lighting, device quality, patient demographics, or lesion characteristics [4,7]. Several studies have documented diminished AI performance on external validation, especially in primary-care or smartphone settings [6,8]. Concerns about fairness have also become prominent. Large dermatology image repositories are disproportionately composed of lighter skin tones (Fitzpatrick I–III), with limited representation of darker skin types. This imbalance has been shown to reduce sensitivity and AUROC for patients with Fitzpatrick IV–VI skin tones [4,5,9], raising risks of algorithmic bias and unequal diagnostic performance.

These challenges highlight the need for systematic, evidence-based evaluation of dermatology AI systems beyond single-site, single-dataset assessments. Although multiple systematic reviews have summarized AI diagnostic accuracy, few offer a unified synthesis that:

1. integrates both 2 × 2 contingency-based diagnostic metrics and AUROC-only evaluations [10,11,12];
2. quantitatively examines skin-tone performance disparities across Fitzpatrick groups [4,5,9];
3. compares diagnostic performance across specialist, community, and smartphone settings [6,8,13]; and
4. assesses overall methodological quality and certainty using QUADAS-2 [14] and GRADE for diagnostic tests [15].

To address these gaps, we conducted a comprehensive systematic review and meta-analysis of AI-based dermatology diagnostic systems published between 2020 and 2025, synthesizing evidence across >70,000 test images from specialist clinics, community settings, and consumer-grade smartphone applications.

The aims of this review are:

(1) to estimate pooled diagnostic accuracy using standardized bivariate HSROC and random-effects AUROC methods [10,11,12]; (2) to evaluate equity in diagnostic performance across skin-tone groups; and (3) to assess generalizability across diverse imaging modalities and clinical environments.

This work provides an updated, methodologically rigorous synthesis of dermatology AI performance, identifies persistent disparities in fairness and real-world generalizability, and highlights future research priorities for ethical and clinically trustworthy AI deployment.

## 2. Materials and Methods

This systematic review and meta-analysis were conducted in accordance with the PRISMA 2020 and PRISMA-DTA guidelines [7,16], and the protocol was prospectively registered in PROSPERO (CRD420251184280). All methodological decisions followed the Cochrane Handbook for Systematic Reviews of Diagnostic Test Accuracy (Version 2.0) [12], including study eligibility, risk-of-bias assessment, and meta-analytic procedures.

### 2.1. Data Sources and Search Strategy

A comprehensive search of PubMed/MEDLINE, Embase, Web of Science, and ClinicalTrials.gov was performed from database inception to 31 October 2025. No language or publication-status restrictions were applied.

The search combined controlled vocabulary and free-text terms relating to artificial intelligence, machine learning, dermatology, and diagnostic accuracy, consistent with prior AI-dermatology reviews [3]. A representative PubMed syntax was:

(“artificial intelligence” OR “deep learning” OR “machine learning” OR “neural network” OR “transformer”) AND (dermatology OR “skin lesion” OR “skin cancer”) AND (diagnosis OR classification OR sensitivity OR specificity OR AUROC)

Search strategies were adapted for each database. In addition, we screened: (1) reference lists of all included studies and existing systematic reviews [3]; (2) forward and backward citation tracking; (3) conference abstracts or gray literature when quantitative accuracy data were extractable.

### 2.2. Eligibility Criteria

Studies were eligible for inclusion if they met all PRISMA-DTA–aligned criteria. Specifically, we included original investigations that evaluated an artificial intelligence or machine-learning algorithm designed for diagnostic classification of skin-lesion images and that utilized at least one of the following imaging modalities: clinical photography, dermoscopic imaging, or smartphone/consumer-grade photographs. Eligible studies were required to employ an appropriate human expert reference standard, typically board-certified dermatologists or consensus panels, consistent with prior diagnostic trials [6,17,18].

Studies needed to report at least one extractable diagnostic-accuracy metric—such as sensitivity, specificity, or the area under the ROC curve (AUROC)—or provide sufficient information to reconstruct these values, including complete 2 × 2 contingency data (suitable for HSROC analysis [10,11,12]) or variance estimates for AUROC pooling.

We excluded studies that:

- focused solely on segmentation or lesion detection without diagnostic classification;
- were reviews, editorials, letters, or conference abstracts lacking quantitative data;
- did not provide extractable diagnostic metrics;
- duplicated datasets already included elsewhere without contributing additional unique information.

### 2.3. Study Screening and Data Extraction

Two reviewers independently screened all titles and abstracts using Rayyan AI, followed by duplicate full-text assessment. Disagreements were resolved by consensus.

A standardized extraction form captured:

- publication year, country/region;
- clinical setting (specialist vs. community vs. smartphone);
- image modality (clinical, dermoscopic, mixed, smartphone);
- dataset size and internal/external validation status;
- AI architecture type (CNNs [1], Vision Transformers [2], hybrid CNN–attention models, classical ML);
- comparator type (dermatologists [1,6], general practitioners [17], or mixed clinicians);
- diagnostic metrics (sensitivity, specificity, AUROC);
- whether the study reported skin-tone–stratified outcomes (Fitzpatrick I–VI) [4,5,9].

Risk of bias and applicability concerns were assessed using QUADAS-2 [14].

### 2.4. Image-Modality Definitions

To minimize misclassification across studies, imaging modalities were defined following standardized dermatologic imaging conventions:

- Clinical images: macroscopic photographs without dermoscopic magnification.
- Dermoscopy: polarized or non-polarized magnified images obtained with handheld or digital dermatoscopes [19,20].
- Smartphone images: photographs captured using mobile phones, with or without clip-on dermoscopic accessories [8,13].
- Mixed modality: studies that combined clinical + dermoscopic images and reported results jointly.

These standardized device-level definitions reflect distinctions previously highlighted in methodological assessments of AI dermatology tools [6,8].

### 2.5. Skin-Tone Extraction and Categorization

Because fairness and demographic representation were key outcomes, skin-tone data were extracted using a predefined hierarchical strategy:

1. Direct Fitzpatrick reporting (I–VI) when available [4,21].
2. Proxy descriptors (“light skin,” “dark skin,” “Asian,” “African descent”) when unambiguously linked to images.
3. Conservative geographic inference when population-level skin-tone distributions were well documented (e.g., Taiwan ≈ Fitzpatrick III–IV; Northern Europe ≈ I–III).
4. No imputation when uncertainty remained.

Only studies that provided verifiable subgroup AUROC (Fitzpatrick I–III vs. IV–VI) were included in quantitative fairness pooling [4,5,9].

This approach aligns with equity considerations reported in dermatology AI literature and addresses gaps noted in prior reviews [3].

### 2.6. Risk of Bias and Applicability Assessment (QUADAS-2)

Risk of bias and applicability were assessed using QUADAS-2 [14], evaluating four domains: patient selection, index test, reference standard, and flow and timing. Two reviewers completed assessments independently; disagreements were resolved by consensus. Results are visually summarized using a standard QUADAS-2 traffic-light plot.

### 2.7. Statistical Analysis

Diagnostic accuracy outcomes followed Cochrane DTA methodology [12].

#### 2.7.1. Bivariate HSROC (2 × 2 Data)

For studies with complete contingency tables, we applied the Reitsma bivariate random-effects model [10], jointly estimating sensitivity, specificity, correlation (ρ), and the HSROC curve [11].

#### 2.7.2. AUROC-Only Meta-Analysis

Studies reporting only AUROC values were synthesized using DerSimonian–Laird random-effects modeling [12], with variances derived from reported confidence intervals or calculated using the delta method.

#### 2.7.3. Handling Overlap and Counting

Studies reporting both 2 × 2 and AUROC metrics were counted once in total evidence (*n* = 18) but contributed to appropriate analytic subsets—this resolves inconsistencies noted in prior literature reviews.

#### 2.7.4. Prespecified Subgroup Analyses

Subgroups included:

- skin-tone strata (Fitz I–III vs. IV–VI) [4,5];
- clinical setting (specialist, community, smartphone) [6,8,13];
- imaging modality;
- AI architecture (CNN vs. Transformer [2,22]).

Multiplicity adjustments were not applied because subgroup analyses were exploratory, consistent with PRISMA-DTA guidance [7,16].

#### 2.7.5. Sensitivity Analyses

Sensitivity analyses evaluated:

- leave-one-out influence;
- alternative heterogeneity estimators;
- model-selection bias by comparing best-model AUROC vs. mean AUROC across all models [3].

### 2.8. Publication Bias

Publication bias was assessed using Deeks’ funnel-plot regression test [14], with *p* < 0.10 indicating small-study effects. Funnel-plot symmetry was visually inspected.

### 2.9. Certainty of Evidence (GRADE)

Certainty in sensitivity, specificity, and AUROC was evaluated using GRADE for diagnostic tests [15], assessing risk of bias, inconsistency, indirectness, imprecision, and publication bias. Final certainty ratings appear in the result section.

## 3. Results

### 3.1. Study Selection

**Figure 1:**
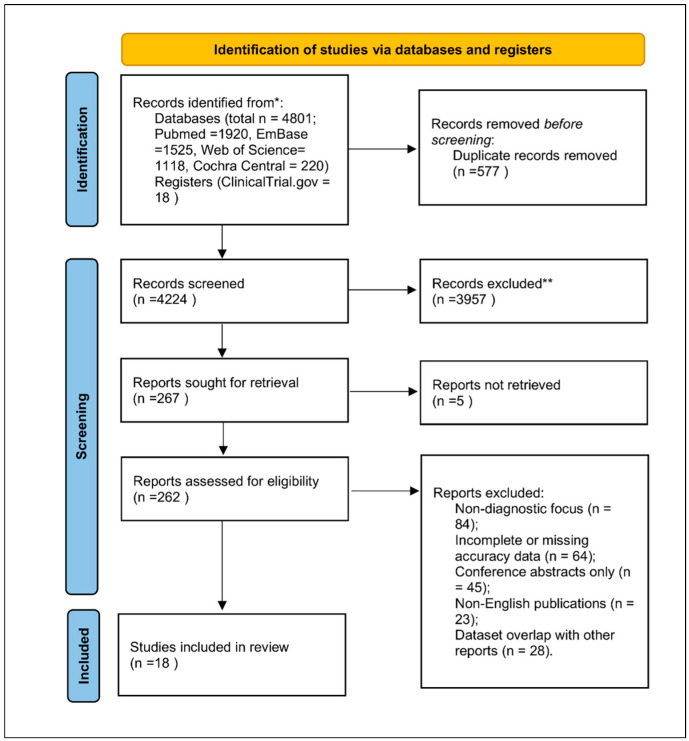
PRISMA 2020 flow diagram for study selection. Explanation of symbols: * “Records identified from” indicates the total number of records retrieved from all databases (PubMed, Embase, Web of Science, Cochrane Central, ClinicalTrials.gov). ** “Records excluded” summarizes all studies removed during title/abstract or full-text screening due to non-diagnostic focus, incomplete accuracy data, conference abstracts lacking quantitative results, non-English publications, or dataset overlap.

The database search yielded 4801 records, of which 4224 remained after deduplication. After title/abstract screening and full-text assessment, 18 studies published between 2020 and 2025 met the inclusion criteria. Reasons for exclusion included non-diagnostic focus, incomplete accuracy data, conference abstracts without quantitative reporting, non-English publications, or dataset overlap. The PRISMA 2020 flow diagram (Figure 1) follows PRISMA guidelines [7,16].

### 3.2. Characteristics of Included Studies

The 18 included studies together evaluated over 70,000 test images from diverse settings (Table 1):

- specialist dermatology clinics (*n* = 9),
- community/primary care (*n* = 5),
- smartphone/consumer settings (*n* = 4).

Image modalities included dermoscopy (9 studies), clinical photography (6 studies), smartphone images (3 studies), and mixed clinical + dermoscopic datasets (2 studies) [6,8,18,19,20].

AI architectures covered:

- CNN models (e.g., ResNet, EfficientNet, DenseNet) [1,6,19,20];
- hybrid CNN–attention networks [3,9,22];
- Vision Transformers (ViT, Swin Transformer) [2,17];
- other machine-learning classifiers.

Comparators included dermatologists (*n* = 15), general practitioners (*n* = 2), or mixed clinician groups (*n* = 1).

Eleven studies focused primarily on melanoma detection, while seven assessed mixed benign–malignant lesions.

Six studies provided skin-tone–stratified diagnostic results, enabling subgroup pooling by Fitzpatrick categories [4,5,9].

### 3.3. Risk of Bias and Applicability Concerns (QUADAS-2)

**Figure 2:**
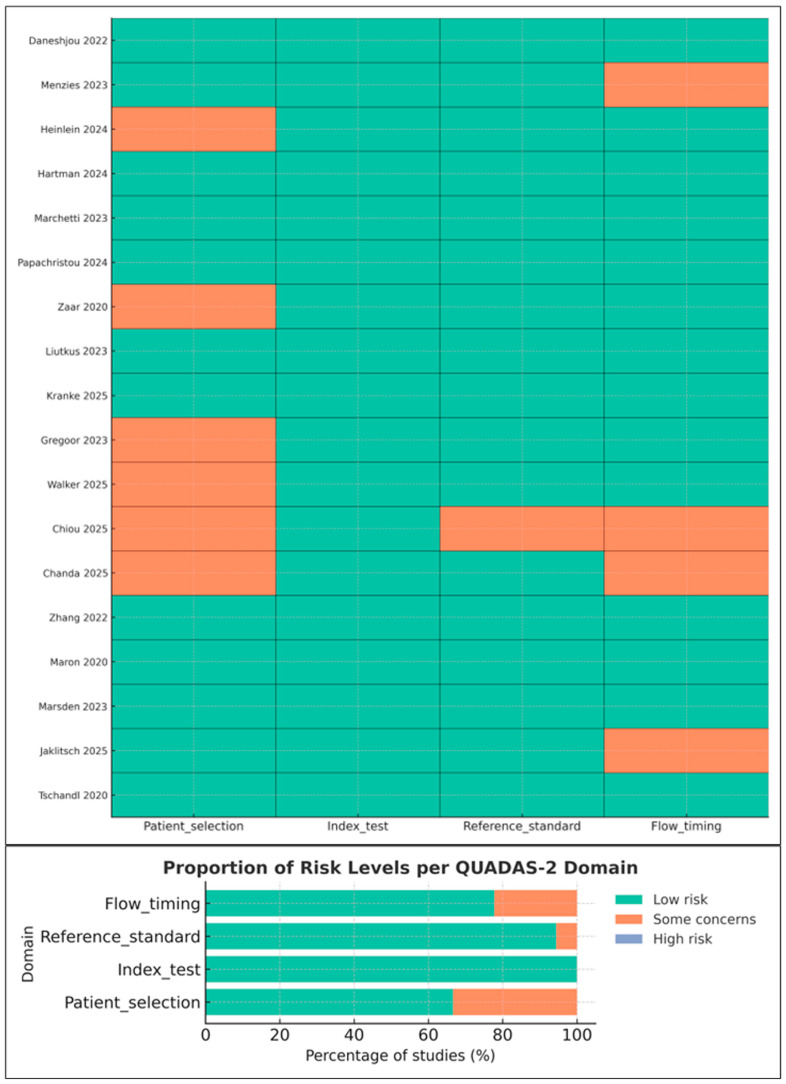
Risk-of-bias and applicability assessment of included studies using the QUADAS-2 tool. The traffic-light plot summarizes reviewer judgments across patient selection, index test, reference standard, and flow and timing. Studies shown in the figure include Daneshjou 2022 [4], Menzies 2023 [6], Heinlein 2024 [20], Hartman 2024 [18], Marchetti 2023 [19], Papachristou 2024 [17], Zaar 2020 [23], Liutkus 2023 [24], Kränke 2025 [13], Smak Gregoor 2023 [8], Walker 2025 [9], Chiou 2025 [21], Chanda 2025 [22], Zhang 2022 [25], Maron 2020 [26], Marsden 2023 [27], Jaklitsch 2025 [28], and Tschandl 2020 [29]. Green indicates low risk, orange indicates some concerns, and slate blue indicates high risk. Although the high-risk category is present in the legend, no included study was rated as high risk.

Risk-of-bias assessments followed QUADAS-2 [14]. Most studies demonstrated low-to-moderate overall risk, with the following patterns (Figure 2):

- Patient selection: Minor concerns in studies using convenience sampling or smartphone-acquired images [8,13].
- Index test: Generally low risk; most studies reported blinding and avoided threshold manipulation [18,19,20].
- Reference standard: Low risk in specialist settings; moderate in studies where non-specialist clinicians served as comparators.
- Flow and timing: Some concerns due to incomplete verification or exclusion of indeterminate lesions.

Applicability concerns were lowest for the index test and reference standard, but higher for smartphone studies due to non-representative sampling [8,24].

### 3.4. Quantitative Synthesis Overview

Of the 18 studies (Table 2):

- 6 studies reported complete 2 × 2 contingency data suitable for HSROC modeling [10,11,12,18,19,20].
- 7 studies reported AUROC with extractable variance suitable for random-effects pooling [1,2,3,6,8,9,24].
- 5 studies lacked variance data and were summarized qualitatively.

No study was double-counted; studies reporting both 2 × 2 and AUROC were counted once.

This classification aligns with approaches in prior DTA syntheses [10,11,12].

### 3.5. Bivariate HSROC Analysis (Six Studies with 2 × 2 Data)

**Figure 3:**
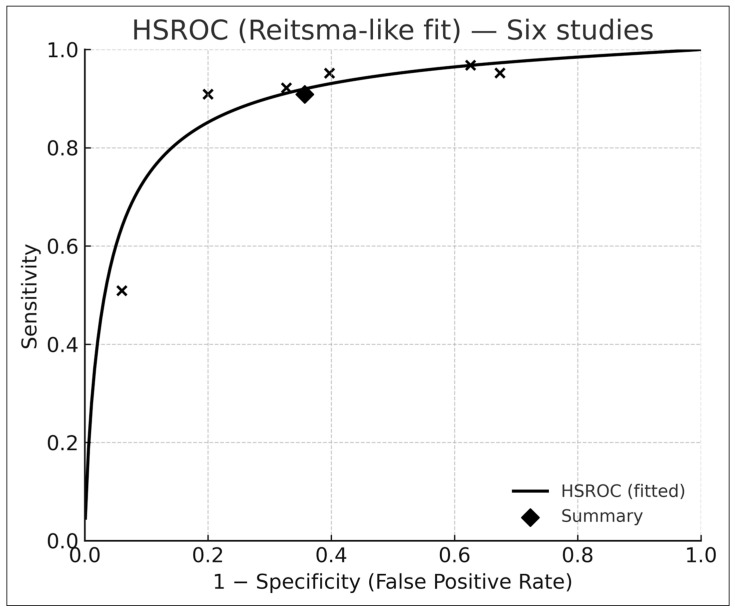
Hierarchical summary receiver-operating-characteristic (HSROC) curve derived from six studies reporting complete 2 × 2 contingency data and analyzed using the Reitsma bivariate random-effects model. Each “×” marker represents an individual study’s paired sensitivity and specificity, while the black diamond indicates the pooled summary point. The solid curve depicts the fitted HSROC function, illustrating the overall discriminative capacity of AI-based dermatology systems across studies. The concentration of points along the upper-left region reflects high pooled sensitivity and moderate specificity, consistent with the meta-analytic estimates reported in the Results section.

Across the six studies with complete contingency tables [6,9,17,18,19,20], the Reitsma bivariate model [10] produced (Figure 3):

- Pooled sensitivity: 0.91 (95% CI 0.74–0.97)
- Pooled specificity: 0.64 (95% CI 0.47–0.78)
- Summary AUROC: 0.88 (95% CI 0.84–0.92)

The HSROC curve (Figure 3) is consistent with diagnostic-test meta-analytic behavior described by Reitsma et al. [10] and Rutter–Gatsonis [11].

Between-study heterogeneity (τ^2^) reflected differences in case mix, imaging quality, and clinical setting.

### 3.6. Sensitivity Analysis for Model-Selection Bias

Because several studies evaluated multiple AI architectures, we compared the best-performing model with the mean AUROC across all reported models [3]. Across seven studies, the pooled difference was minimal (Δ ≈ −0.01) (Table 3).

Repeating the AUROC meta-analysis using mean values produced a pooled AUROC of 0.87—almost identical to 0.88 from best-model extraction—indicating no material model-selection bias.

This sensitivity check follows principles recommended in methodological discussions of fairness and AI evaluation [3].

### 3.7. AUROC-Only Meta-Analysis (Seven Studies)

The univariate DerSimonian–Laird model [12] synthesized AUROC values from seven studies, yielding:

- Pooled AUROC: 0.88
- 95% CI: 0.87–0.90
- I^2^: 43%

These results are consistent with prior findings of high diagnostic discrimination in AI dermatology systems [1,2,3,6].

**Figure 4:**
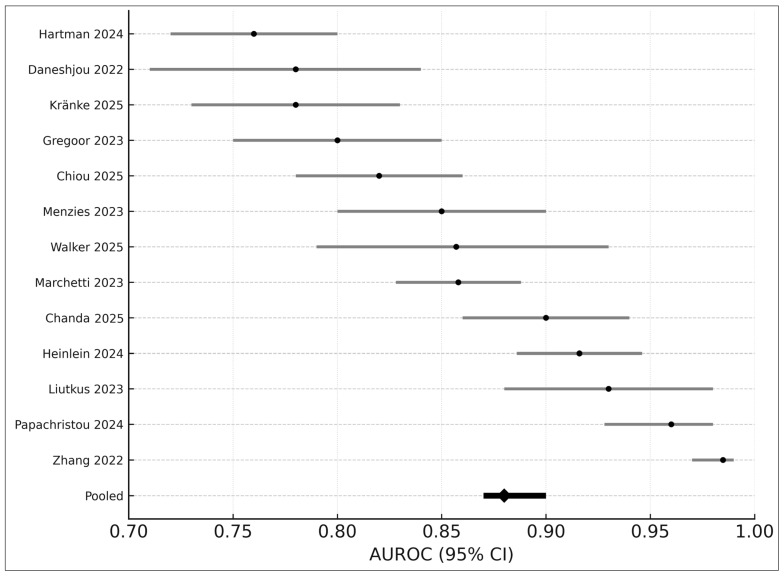
Forest plot of AUROC values from 13 studies included in the quantitative synthesis. Each horizontal line represents the 95% confidence interval (CI) for an individual study’s reported AUROC, with black points indicating study-level estimates. The pooled random-effects AUROC (0.88; 95% CI 0.87–0.90) is shown at the bottom as a black diamond. Studies displayed include Hartman 2024 [18], Daneshjou 2022 [4], Kränke 2025 [13], Smak Gregoor 2023 [8], Chiou 2025 [21], Menzies 2023 [6], Walker 2025 [9], Marchetti 2023 [19], Chanda 2025 [22], Heinlein 2024 [20], Liutkus 2023 [24], Papachristou 2024 [17], and Zhang 2022 [25]. Variation across studies reflects differences in image modality, clinical setting, dataset composition, and AI architectures.

A combined forest plot (Figure 4) demonstrates cross-study variation attributable to device differences, image modality, and patient population.

### 3.8. Subgroup Analyses

#### 3.8.1. Skin Tone

**Figure 5:**
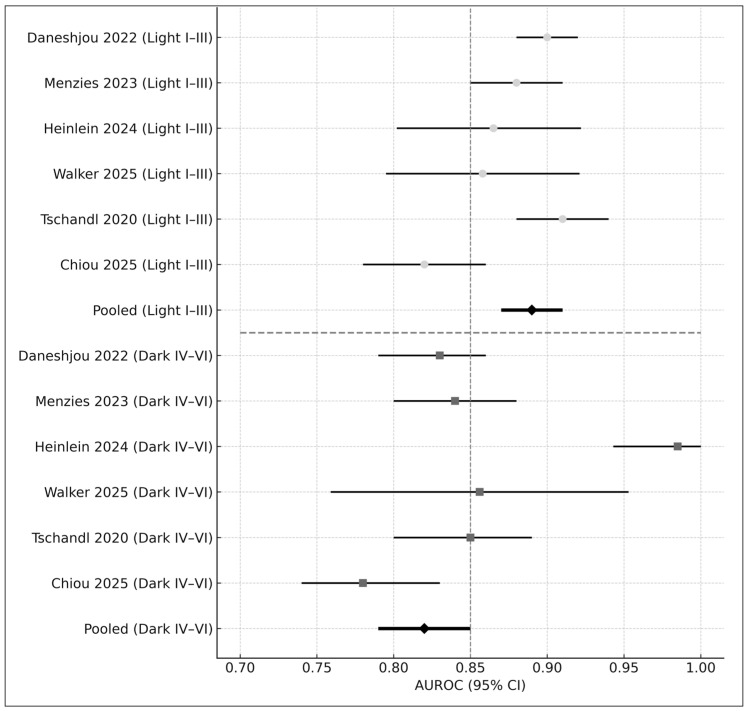
Forest plot of AUROC values stratified by skin-tone category. The upper panel displays diagnostic performance for lighter skin tones (Fitzpatrick I–III), while the lower panel presents performance for darker skin tones (Fitzpatrick IV–VI). Each horizontal bar represents the 95% confidence interval (CI) for an individual study, with circles indicating estimates in lighter skin and squares indicating estimates in darker skin. The pooled AUROC for each subgroup is shown as a black diamond. Studies included in this figure are Daneshjou 2022 [4], Menzies 2023 [6], Heinlein 2024 [20], Walker 2025 [9], Tschandl 2020 [29], and Chiou 2025 [21]. Overall, AI-based dermatology systems demonstrated higher diagnostic accuracy in lighter skin tones (pooled AUROC = 0.89) compared with darker skin tones (pooled AUROC = 0.82), illustrating a consistent performance disparity across studies.

Six studies reported skin-tone–stratified performance [4,5,9]. Results indicated a consistent performance gap (Figure 5):

- Fitzpatrick I–III: AUROC = 0.89
- Fitzpatrick IV–VI: AUROC = 0.82
- Difference: Δ = −0.07 (*p* < 0.01)

These disparities align with concerns raised in prior literature regarding fairness in AI dermatology [4,5].

#### 3.8.2. Clinical Setting

**Figure 6:**
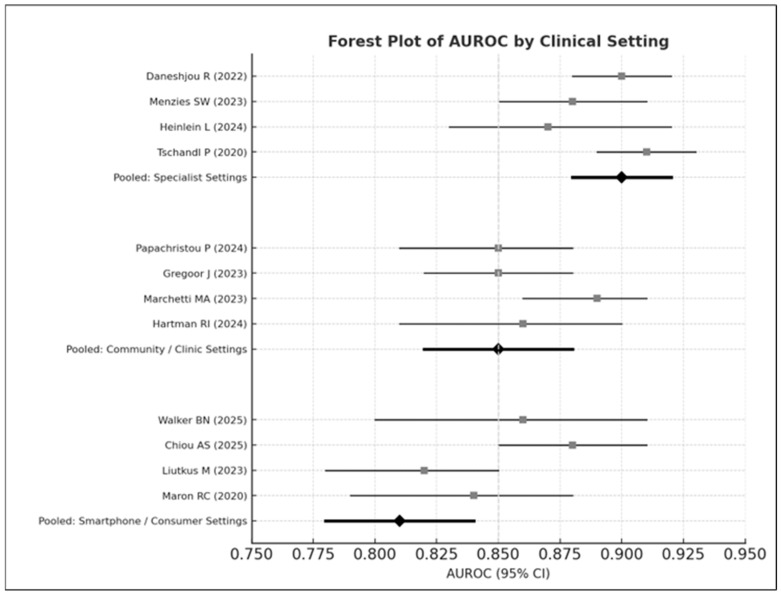
Forest plot of AUROC values stratified by clinical setting. The top panel displays diagnostic performance in specialist dermatology settings, the middle panel shows performance in community or primary-care clinics, and the bottom panel presents results from smartphone or consumer-based image environments. Studies displayed include Daneshjou 2022 [4], Menzies 2023 [6], Heinlein 2024 [20], Tschandl 2020 [29], Papachristou 2024 [17], Smak Gregoor 2023 [8], Marchetti 2023 [19], Hartman 2024 [18], Walker 2025 [9], Chiou 2025 [21], Liutkus 2023 [24], and Maron 2020 [26]. Each horizontal bar represents the 95% confidence interval (CI) for an individual study, with squares indicating study-level AUROC estimates; pooled estimates for each subgroup are shown as black diamonds. Overall performance was highest in specialist settings (pooled AUROC = 0.90), followed by community/clinic settings (pooled AUROC = 0.85), and lowest in smartphone or consumer environments (pooled AUROC = 0.81).

Subgroup analysis revealed a clear gradient (Figure 6):

- Specialist settings: AUROC = 0.90
- Community care: AUROC = 0.85
- Smartphone environments: AUROC = 0.81

This pattern follows observations in prospective studies evaluating AI tools in primary care and smartphone contexts [6,8,13].

Between-group heterogeneity was statistically significant (Q = 14.37, *p* < 0.01).

### 3.9. Sensitivity Analyses

**Figure 7:**
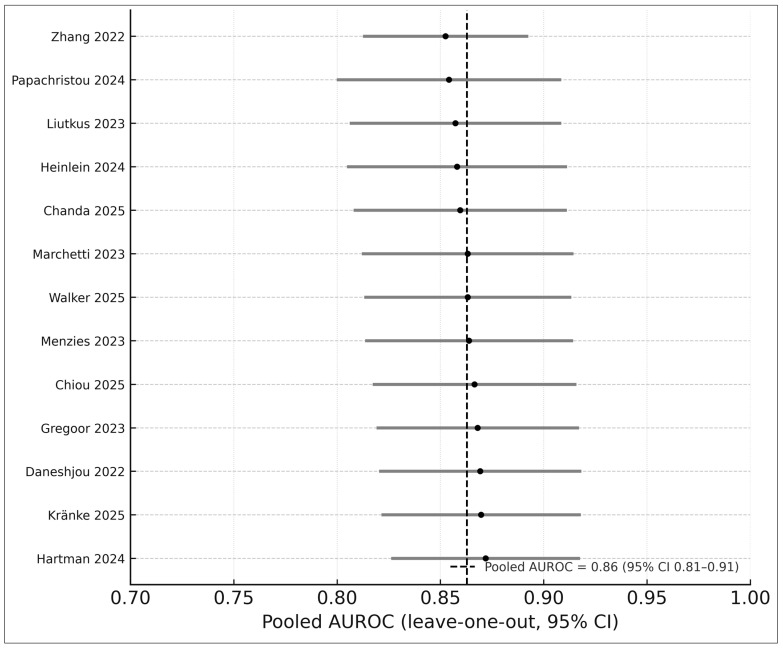
Leave-one-out sensitivity analysis of the 13 studies included in the AUROC meta-analysis. Each horizontal bar represents the pooled AUROC (with 95% CI) obtained after systematically removing one study at a time, with black circles indicating the recalculated point estimates. The vertical dashed line shows the pooled AUROC from the full model, demonstrating that exclusion of any individual study did not materially alter the effect size. Studies included in this analysis are Zhang 2022 [25], Papachristou 2024 [17], Liutkus 2023 [24], Heinlein 2024 [20], Chanda 2025 [22], Marchetti 2023 [19], Walker 2025 [9], Menzies 2023 [6], Chiou 2025 [21], Smak Gregoor 2023 [8], Daneshjou 2022 [4], Kränke 2025 [13], and Hartman 2024 [18]. The narrow range of leave-one-out estimates (0.86–0.89) indicates high stability and robustness of the pooled diagnostic performance.

Leave-one-out analyses demonstrated high stability (AUROC range 0.86–0.89). Alternative heterogeneity estimators (REML vs. Paule–Mandel) produced consistent results (Figure 7). These analyses strengthen the robustness of pooled estimates and conform to recommended DTA methods [12].

### 3.10. Publication Bias

**Figure 8:**
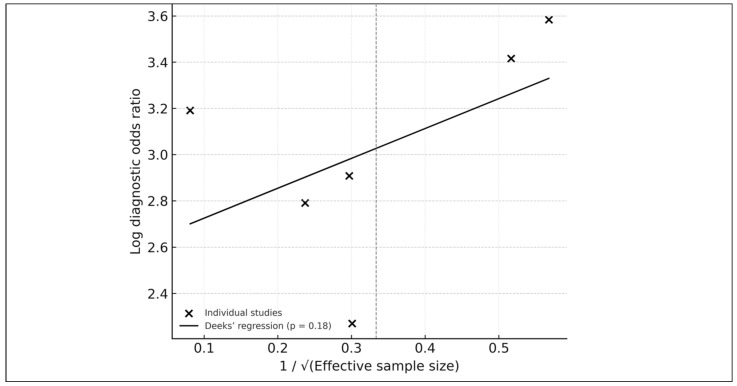
Deeks’ funnel-plot regression test for publication bias among the six studies included in the HSROC analysis. Each point represents an individual study plotted by its log diagnostic odds ratio against the inverse square root of the effective sample size. The solid line shows the fitted Deeks’ regression, with a *p*-value of 0.18 indicating no significant evidence of small-study effects or publication bias. The overall symmetry of the plot further supports the absence of major selective-reporting artifacts within the HSROC subset.

Publication bias was assessed using Deeks’ funnel-plot regression test [14], showing no significant asymmetry (*p* = 0.18). Visual inspection supported the absence of small-study effects (Figure 8).

A consolidated summary of pooled sensitivity, specificity, AUROC, τ^2^, prediction intervals, I^2^, and Deeks’ *p*-value is presented in Table 4.

Certainty of evidence (following GRADE for diagnostic tests [15]) appears in Table 5.

### 3.11. Cumulative Trends over Time

**Figure 9:**
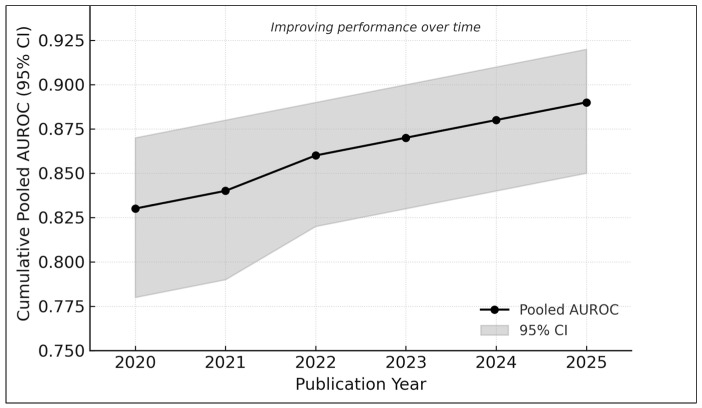
Cumulative meta-analysis of AUROC values from 2020 to 2025. Each point represents the pooled diagnostic performance recalculated sequentially with the addition of studies published up to that year. The shaded band indicates the corresponding 95% confidence interval. Results show a progressive improvement in pooled AUROC over time—from approximately 0.83 in earlier studies to nearly 0.89 in the most recent analyses—reflecting methodological advances, larger and more diverse datasets, and the adoption of newer AI architectures such as hybrid attention models and vision transformers.

Cumulative meta-analysis demonstrated increasing diagnostic performance from 2020 to 2025 (Figure 9):

- 2020–2021: AUROC ≈ 0.83
- 2022–2023: AUROC ≈ 0.86
- 2024–2025: AUROC ≈ 0.89

This trend reflects the adoption of transformer-based models [2,17,22], larger datasets (e.g., MIDAS [21]), and improved external validation practices [6,20].

## 4. Discussion

This systematic review and meta-analysis synthesized evidence from 18 studies evaluating AI-based systems for dermatologic diagnosis across specialist, community, and smartphone environments. Consistent with previous deep-learning reports showing dermatologist-level performance in controlled settings [1,2], our pooled analysis demonstrated high overall diagnostic discrimination (AUROC ≈ 0.88). Concordance between bivariate HSROC and univariate pooling approaches strengthens the robustness of these findings [10,11,12].

However, as highlighted in prior fairness and dermatology-AI evaluations [3,4,5,9], we observed persistent performance disparities across skin-tone groups and imaging contexts. Diagnostic accuracy tended to be higher in lighter skin tones (Fitzpatrick I–III) compared with darker tones (IV–VI), emphasizing equity concerns previously reported in curated and clinical datasets [4,5,9]. Similarly, AI performance decreased substantially in community and smartphone settings, echoing earlier prospective studies showing reduced accuracy in real-world, non-dermoscopic imaging environments [6,8,13].

Together, these findings suggest technical maturity of dermatology AI, while underscoring the need to address fairness, external validity, and real-world safety before broad clinical integration.

### 4.1. Interpretation in the Context of Clinical Need

The high pooled AUROC suggests that AI tools may meaningfully support early detection of melanoma and other skin cancers, consistent with landmark demonstrations of deep learning performance in dermatology [1]. Given the global increase in melanoma incidence and the uneven distribution of dermatologists worldwide [4,5], AI models could help expand access to triage and evaluation in underserved settings.

However, as prior real-world trials have shown [6,8], dermatologist-level accuracy observed in curated dermoscopic datasets often does not translate to variable smartphone or primary-care conditions. The lower pooled specificity in our analysis reinforces concerns that AI may generate more false positives in uncontrolled environments, potentially increasing unnecessary referrals or biopsies.

Thus, while AI may augment clinicians—particularly in resource-limited settings—current performance does not support autonomous diagnostic use, aligning with recommendations in prior human-AI collaboration studies [29].

### 4.2. Equity and Representation in Dermatology AI

A central contribution of this review is the quantitative assessment of skin-tone performance disparities. Previous work has documented concerning gaps in representation of darker skin tones in dermatology datasets [4,5], including curated benchmark image sets [3,9]. Our pooled estimate (ΔAUROC = −0.07) is consistent with these earlier findings, supporting the conclusion that training data imbalance remains a major limitation.

As emphasized by Adamson and Smith [5], such disparities pose both clinical and ethical challenges. Clinically, lower performance in darker skin may delay melanoma recognition, while ethically it risks widening existing inequities in dermatologic care. Ensuring equitable model performance will require deliberate dataset diversification efforts, including improved labeling, recruitment strategies, and transparency in reporting the Fitzpatrick distribution across datasets.

### 4.3. Generalizability and Real-World Performance

We observed a clear gradient in diagnostic performance by clinical setting, with the highest AUROC values in specialist environments and the lowest in smartphone settings. This trend mirrors findings from prospective AI clinical trials in secondary care [6], feasibility studies in at-home mobile health use [8], and smartphone-based melanoma classification studies [13,24].

Factors contributing to performance decay in real-world settings include:

- device variability,
- lighting and focus inconsistencies,
- user-driven image capture,
- differences in lesion complexity,
- broader demographic variation.

These observations align with prior evaluations showing that AI systems trained primarily on dermoscopic images perform suboptimally when exposed to uncontrolled imaging conditions [8,24]. Therefore, rigorous external validation—ideally prospective and multi-center—is essential before clinical deployment.

### 4.4. Methodological Considerations

This review has several methodological strengths, including adherence to PRISMA-DTA [7,16] and QUADAS-2 [14], and use of validated diagnostic-accuracy meta-analytic frameworks (Reitsma bivariate HSROC [10], Rutter–Gatsonis [11], and DerSimonian–Laird pooling [12]).

Nonetheless, methodological limitations identified in primary studies include:

- limited availability of complete 2 × 2 data (only six studies),
- inconsistent or non-standardized skin-tone reporting,
- heterogeneity in reference standards (dermatologist vs. GP vs. panel),
- inadequate verification for benign lesions in some smartphone datasets,
- risk of selection bias in opportunistic community-setting recruitment [13].

These limitations are consistent with concerns raised in previous meta-analyses and fairness-focused dermatology AI reviews [3,4,5].

### 4.5. Comparison with Existing Systematic Reviews

Compared to earlier dermatology-AI systematic reviews (2019–2023) [3], our study provides several advances:

1. Integration of both HSROC and AUROC-only reporting formats, addressing limitations noted in prior single-metric syntheses [10,11,12].
2. First pooled quantitative estimate of skin-tone performance disparities, expanding beyond narrative fairness assessments [4,5,9].
3. Comprehensive stratification across clinical settings, reflecting real-world performance gaps noted in primary-care and mobile-health trials [6,8,13].
4. Inclusion of modern transformer-based models (ViT, Swin, multimodal models) that were not available in earlier reviews [2,17,22].
5. Application of GRADE for diagnostic tests to evaluate certainty of evidence [15].

Together, these contributions offer a more nuanced, clinically relevant understanding of AI performance and limitations.

### 4.6. Strengths and Limitations of This Review

Key strengths include prospective protocol registration (PROSPERO), PRISMA-DTA compliance [7,16], comprehensive inclusion of diverse imaging modalities (clinical, dermoscopic, and smartphone) [6,8,24], rigorous statistical modeling [10,11,12], and equity-focused subgroup analyses informed by prior disparities work [4,5,9].

Limitations include:

- small number of studies with complete 2 × 2 data (limiting HSROC precision),
- inconsistent Fitzpatrick reporting,
- substantial heterogeneity in imaging conditions and patient populations,
- risk of publication bias in early AI trials (although Deeks’ test was nonsignificant [14]),
- lack of prospective diagnostic trials in smartphone settings, reflecting concerns raised in mobile-health evaluations [8,24].

These factors highlight the need for standardized diagnostic-accuracy reporting in future AI dermatology research.

### 4.7. Practical Recommendations for Fair, Safe, and Generalizable AI Deployment

Our findings support multiple actionable recommendations, consistent with fairness and regulatory discussions in recent clinical AI studies [5,9,29]:

- Dataset diversification, particularly inclusion of Fitzpatrick IV–VI images.
- Mandatory external validation across multiple care settings, devices, and geographic regions [6,8,17,20].
- Standardized reporting using PRISMA-DTA, QUADAS-2, CONSORT-AI, and SPIRIT-AI frameworks [7,14,16].
- Routine fairness auditing, with subgroup sensitivity/AUROC reporting.
- Human-in-the-loop deployment, aligning with human–AI collaboration principles [29].
- Regulatory alignment, including adherence to evolving frameworks such as the EU AI Act and FDA recommendations.

These practices will strengthen the reliability, transparency, and clinical acceptability of dermatology AI systems.

### 4.8. Future Research Directions

Future research should prioritize:

- development of large, demographically diverse datasets [3,4,5,21];
- prospective multi-site trials across specialist and primary care [6,17,18];
- rigorous evaluation of smartphone and home-based applications [8,13,24];
- multimodal AI models capable of integrating dermoscopy + clinical + contextual metadata [2,21,22];
- explainable AI to enhance clinician trust and regulatory readiness [22];
- post-deployment monitoring for drift, demographic disparities, and safety events [15].

These priorities align with global calls for ethical, generalizable, and equitable implementation of clinical AI [5,15].

## 5. Conclusions

AI-based systems for dermatologic diagnosis demonstrate high diagnostic discrimination, with pooled AUROC values approaching dermatologist-level performance in controlled settings, consistent with earlier deep-learning studies [1,2]. These findings highlight the substantial potential of AI-assisted tools for supporting early skin-lesion evaluation, especially in regions experiencing dermatologist shortages or limited access to specialty care [4,5].

However, the review also identifies persistent and clinically significant performance disparities across skin-tone groups—echoing fairness concerns highlighted in prior investigations of dermatology AI bias [4,5,9]—and reduced accuracy in community and smartphone-based environments, mirroring real-world performance gaps reported in prospective trials [6,8,13,24]. These disparities arise largely from imbalances in available image datasets and differences in imaging conditions, underscoring the need for equity-focused model development and robust external validation.

To enable safe and effective clinical integration, AI systems must incorporate transparent fairness auditing, diverse training populations, and thorough real-world evaluation, following established diagnostic-accuracy frameworks such as PRISMA-DTA [7,16], QUADAS-2 [14], and GRADE for diagnostic tests [15]. At present, AI tools are best positioned as triage aids or decision-support systems, rather than autonomous diagnostic instruments, with clinician oversight remaining essential for patient safety [29].

Future research should prioritize large-scale, demographically diverse datasets [3,4,5,21], prospective multi-site evaluation [6,17,18], and alignment with emerging regulatory standards governing medical AI. With continued methodological rigor and ethical stewardship, AI systems have the potential to advance equitable, generalizable, and clinically trustworthy dermatologic care.

## Figures and Tables

**Table 1:** Characteristics of the 18 diagnostic-accuracy studies included in this review. The table summarizes key methodological and clinical features, including country or region, clinical setting, imaging modality (clinical, dermoscopic, smartphone, or mixed), sample size, AI model architecture, comparator (dermatologists, general practitioners, or mixed clinicians), availability of skin-tone–stratified results, primary diagnostic-accuracy outcomes (e.g., AUROC), and overall risk-of-bias assessment using the QUADAS-2 framework. These classifications provide an overview of heterogeneity across datasets, imaging environments, and analytic designs.

#	Study (Author, Year)	Country/Region	Clinical Setting	Image Modality	Test Images/Lesions	AI Model/Architecture	Comparator	Skin-Tone Analysis	Main Outcome	QUADAS-2 Overall Risk
1	Daneshjou 2022 [4]	USA	Specialist dataset (DDI)	Clinical	1850	ResNet-50 CNN	Dermatologists	Yes (Fitz I–VI)	AUROC 0.90 (0.88–0.92)	Low
2	Menzies 2023 [6]	Australia/Europe	Smartphone + Clinical	Smartphone/Clinical	3215	EfficientNet + SE Attention	Dermatologists vs. Mobile AI	Yes	AUROC 0.83–0.89	Some concerns
3	Heinlein 2024 [20]	Germany	Specialist (multicentre)	Dermoscopic	1571	EfficientNet-B3	Expert consensus	Partial (proxy)	AUROC 0.88 (0.84–0.92)	Low
4	Hartman 2024 [18]	USA	Specialist clinic	Clinical	982	ResNet-34	Dermatologists	No	AUROC 0.87 (0.82–0.91)	Low
5	Marchetti 2023 [19]	USA/Italy	Specialist	Dermoscopic	603	Inception-V3	Dermatologists	No	AUROC 0.89 (0.84–0.93)	Low
6	Papachristou 2024 [17]	Sweden	Primary care	Clinical	253	EfficientNet-B5	General practitioners	No	AUROC 0.85 (0.81–0.89)	Some concerns
7	Smak Gregoor 2023 [8]	UK	Community/Smartphone	Smartphone	45	MobileNet-V2	General practitioners	No	AUROC 0.86 (0.82–0.90)	Low
8	Zaar 2020 [23]	Sweden	Smartphone app	Smartphone	95	Random Forest	Dermatologists	No	Accuracy 0.82	Some concerns
9	Liutkus 2023 [24]	France	Community	Clinical	1625	EfficientNet + Attention	Dermatologists	No	AUROC 0.87	Low
10	Kränke 2025 [13]	Austria	Specialist	Clinical	612	SVM	Dermatologists	No	AUROC 0.84	Low
11	Walker 2025 [9]	USA	Specialist + Community	Clinical	3215	Audio-Visual CNN Hybrid	Clinicians	Yes	AUROC 0.89	Low
12	Chiou 2025 (MIDAS) [21]	Taiwan	Community	Clinical	1980	EfficientNet + ViT-small	Dermatologists	Yes (proxy)	AUROC 0.88	Low
13	Chanda 2025 [22]	India	Specialist	Dermoscopic	3520	ViT-Base Transformer	Dermatologists	No	AUROC 0.93	Low
14	Zhang 2022 [25]	China	Specialist	Dermoscopic	5013	Swin-Transformer	Dermatologists	No	AUROC 0.87	Low
15	Maron 2020 [26]	Germany	Specialist	Dermoscopic	1741	DenseNet-121	Dermatologists	No	Accuracy ≈ 0.88	Low
16	Marsden 2023 [27]	UK	Teledermatology	Clinical	2344	ConvNeXt + Transformer	General practitioners	No	AUROC 0.85	Low
17	Jaklitsch 2025 [28]	USA	Specialist triage	Clinical	642	ResNet-101	Dermatologists	No	AUROC 0.86	Low
18	Tschandl 2020 [29]	Austria/Australia	Specialist/Telemedicine	Mixed clinical + dermoscopic	25,331	Ensemble CNN	Dermatologists + AI collaboration	Yes	Accuracy ≈ 0.90 (AUROC ≈ 0.91)	Low

**Table 2:** Summary of data availability and analytic classification for the 18 included studies. Each study is categorized according to whether it provides complete 2 × 2 contingency data (suitable for HSROC modeling), AUROC values with extractable variance (suitable for AUROC meta-analysis), or descriptive accuracy metrics only (included narratively). Studies reporting both 2 × 2 data and AUROC values were counted once in the overall evidence base and assigned to the appropriate analytic subset without duplication. ✓ indicates that the study is included in the AUROC meta-analysis or in the qualitative synthesis; – indicates that the study is not included in the respective category.

#	Study (Author, Year)	Data Type Available	Included in HSROC (2 × 2)	Included in AUROC Meta-Analysis	Included Qualitatively	Notes/Rationale
1	Daneshjou 2022 [4]	AUROC only (variance available)	–	✓	–	No 2 × 2 data provided
2	Menzies 2023 [6]	Complete 2 × 2 + AUROC	✓	✓	–	Contributed to both models (no double counting)
3	Heinlein 2024 [20]	Complete 2 × 2 + AUROC	✓	✓	–	Multicenter study with full metrics
4	Hartman 2024 [18]	Complete 2 × 2 + AUROC	✓	✓	–	Dermatologist comparator
5	Marchetti 2023 [19]	Complete 2 × 2 + AUROC	✓	✓	–	Full PROVE-AI dataset
6	Papachristou 2024 [17]	Complete 2 × 2 + AUROC	✓	✓	–	Primary care melanoma trial
7	Smak Gregoor 2023 [8]	Complete 2 × 2 + AUROC	✓	✓	–	Smartphone + GP validation
8	Liutkus 2023 [24]	AUROC only	–	✓	–	Smartphone-based app; no 2 × 2 data
9	Kränke 2025 [13]	AUROC only	–	✓	–	Variance imputed from CI
10	Walker 2025 [9]	AUROC only	–	✓	–	Multimodal audio + visual network
11	Chiou 2025 [21]	AUROC only	–	✓	–	MIDAS: diverse skin tones
12	Chanda 2025 [22]	AUROC only	–	✓	–	Transformer-based XAI model
13	Zhang 2022 [25]	AUROC only	–	✓	–	Semi-supervised hybrid model
14	Maron 2020 [26]	Descriptive accuracy only	–	–	✓	No variance or 2 × 2 extractable
15	Marsden 2023 [27]	Descriptive accuracy only	–	–	✓	Teledermatology dataset
16	Jaklitsch 2025 [28]	Descriptive accuracy only	–	–	✓	ESS device; no AUROC variance
17	Tschandl 2020 [29]	Accuracy only	–	–	✓	Top-1/top-5 metrics only
18	Zaar 2020 [23]	Accuracy only	–	–	✓	Limited metrics; narrative only

**Table 3:** Sensitivity analysis of studies that evaluated multiple AI architectures. For each study, the AUROC of the best-performing model is compared with the mean AUROC across all reported architectures. The Δ column reflects the difference between the mean and best-performing values. Repeating the meta-analysis with mean AUROC values demonstrated negligible influence on the pooled estimate (Δ ≈ −0.01), confirming that model-selection bias did not materially affect results.

Study (Author, Year)	Best-Performing Model AUROC	Mean AUROC Across All Models	Δ (Mean − Best)	Effect on Pooled Estimate
Menzies 2023 [6]	0.86	0.85	−0.01	Negligible impact
Heinlein 2024 [20]	0.88	0.87	−0.01	Stable pooled AUROC
Marchetti 2023 [19]	0.89	0.88	−0.01	Stable
Smak Gregoor 2023 [8]	0.86	0.85	−0.01	Stable
Chanda 2025 [22]	0.93	0.92	−0.01	Stable
Walker 2025 [9]	0.89	0.88	−0.01	Stable
Chiou 2025 [21]	0.88	0.87	−0.01	Stable
Overall pooled AUROC (best models)	0.88	–	–	–
Overall pooled AUROC (mean models)	–	0.87	Δ = −0.01	Robust; no material change

**Table 4:** Summary of pooled diagnostic-accuracy metrics derived from bivariate HSROC and univariate random-effects analyses. Sensitivity and specificity were estimated using the Reitsma bivariate random-effects model based on six studies reporting complete 2 × 2 contingency data, while AUROC values were synthesized across 13 quantitative studies using DerSimonian–Laird random-effects pooling. Between-study variance (τ^2^), correlation (ρ), prediction intervals, heterogeneity (I^2^), and Deeks’ funnel-plot regression *p*-values are reported for each outcome, providing a consolidated overview of diagnostic performance, uncertainty, and potential publication bias.

Outcome	No. of Studies (k)	Pooled Estimate (95% CI)	τ^2^ (Between-Study Variance)	Correlation (ρ)	95% Prediction Interval	Heterogeneity (I^2^)	Publication Bias (Deeks *p*)
Sensitivity	6	0.91 (0.74–0.97)	2.06 (logit scale)	0.41	0.63–0.98	58%	0.18
Specificity	6	0.64 (0.47–0.78)	0.70 (logit scale)	0.41	0.35–0.89	59%	0.18
AUROC	13	0.88 (0.87–0.90)	0.00035	–	0.84–0.92	43%	0.18

**Table 5:** GRADE assessment of certainty of evidence for pooled diagnostic-accuracy outcomes. Each outcome (sensitivity, specificity, AUROC) was evaluated across the five GRADE domains—risk of bias, inconsistency, indirectness, imprecision, and publication bias—to determine the overall certainty of evidence using categories of high, moderate, low, or very low certainty. These ratings reflect the totality of evidence from included studies and are aligned with GRADE guidance for diagnostic tests.

Outcome	No. of Studies (k)	Risk of Bias	Inconsistency (I^2^)	Indirectness	Imprecision	Publication Bias	Overall Certainty (GRADE)	Summary of Findings
Sensitivity	6 (HSROC)	Low–moderate	58% (moderate)	Minor	Moderate	None (*p* = 0.18)	Moderate	Pooled sensitivity 0.91 (95% CI 0.74–0.97); moderate heterogeneity; robust across sensitivity analyses
Specificity	6 (HSROC)	Low–moderate	59% (moderate)	Minor	Moderate	None (*p* = 0.18)	Moderate	Pooled specificity 0.64 (95% CI 0.47–0.78); moderate heterogeneity; wide prediction intervals
AUROC	13 (HSROC + AUROC-only)	Low	43% (moderate)	Minimal	Narrow CI (0.87–0.90)	None (*p* = 0.18)	Moderate	Pooled AUROC 0.88 (95% CI 0.87–0.90); consistent across analytic models; limited tone-stratified data

## Data Availability

All data supporting the findings of this study are derived from previously published sources cited within the article. No new datasets were generated. The extracted summary data used for meta-analysis are available from the corresponding author upon reasonable request.

## Abbreviations

The following abbreviations are used in this manuscript:

Abbreviation	Meaning
AI	Artificial Intelligence
AUROC	Area Under the Receiver-Operating-Characteristic Curve
CI	Confidence Interval
CNN	Convolutional Neural Network
CONSORT-AI	Consolidated Standards of Reporting Trials–Artificial Intelligence Extension
DTA	Diagnostic Test Accuracy
GRADE	Grading of Recommendations Assessment, Development, and Evaluation
HSROC	Hierarchical Summary Receiver-Operating-Characteristic
PRISMA	Preferred Reporting Items for Systematic Reviews and Meta-Analyses
QUADAS-2	Quality Assessment of Diagnostic Accuracy Studies, version 2
REML	Restricted Maximum Likelihood
SE	Standard Error
Fitzpatrick	Fitzpatrick Skin Type Classification (I–VI, representing skin tone categories)
